# Dehydrogenation Mechanism of Three Stereoisomers of Butane-2,3-Diol in *Pseudomonas putida* KT2440

**DOI:** 10.3389/fbioe.2021.728767

**Published:** 2021-08-26

**Authors:** Yidong Liu, Xiuqing Wang, Liting Ma, Min Lü, Wen Zhang, Chuanjuan Lü, Chao Gao, Ping Xu, Cuiqing Ma

**Affiliations:** ^1^ State Key Laboratory of Microbial Technology, Shandong University, Qingdao, China; ^2^ Center for Gene and Immunotherapy, The Second Hospital of Shandong University, Jinan, China; ^3^ State Key Laboratory of Microbial Metabolism, Joint International Research Laboratory of Metabolic and Developmental Sciences, School of Life Sciences and Biotechnology, Shanghai Jiao Tong University, Shanghai, China

**Keywords:** butane-2,3-diol, *Pseudomonas putida* KT2440, (2*R*,3*R*)-butane-2,3-diol dehydrogenase, quinoprotein alcohol dehydrogenase, lanthanide

## Abstract

*Pseudomonas putida* KT2440 is a promising chassis of industrial biotechnology due to its metabolic versatility. Butane-2,3-diol (2,3-BDO) is a precursor of numerous value-added chemicals. It is also a microbial metabolite which widely exists in various habiting environments of *P. putida* KT2440. It was reported that *P. putida* KT2440 is able to use 2,3-BDO as a sole carbon source for growth. There are three stereoisomeric forms of 2,3-BDO: (2*R*,3*R*)-2,3-BDO, *meso*-2,3-BDO and (2*S*,3*S*)-2,3-BDO. However, whether *P. putida* KT2440 can utilize three stereoisomeric forms of 2,3-BDO has not been elucidated. Here, we revealed the genomic and enzymic basis of *P. putida* KT2440 for dehydrogenation of different stereoisomers of 2,3-BDO into acetoin, which will be channeled to central mechanism via acetoin dehydrogenase enzyme system. (2*R*,3*R*)-2,3-BDO dehydrogenase (PP0552) was detailedly characterized and identified to participate in (2*R*,3*R*)-2,3-BDO and *meso*-2,3-BDO dehydrogenation. Two quinoprotein alcohol dehydrogenases, PedE (PP2674) and PedH (PP2679), were confirmed to be responsible for (2*S*,3*S*)-2,3-BDO dehydrogenation. The function redundancy and inverse regulation of PedH and PedE by lanthanide availability provides a mechanism for the adaption of *P. putida* KT2440 to variable environmental conditions. Elucidation of the mechanism of 2,3-BDO catabolism in *P. putida* KT2440 would provide new insights for bioproduction of 2,3-BDO-derived chemicals based on this robust chassis.

## Introduction

*Pseudomonas putida* is known for its rapid growth, low nutrient demand, and adaption to various physicochemical stresses (Nikel and de Lorenzo, 2018). *P. putida* KT2440, the first Gram-negative soil bacterium which was certified as generally recognized as safe (i.e., GRAS) (Nelson, 2002; Nelson et al., 2002), has attracted substantial attention as a new workhorse for bioindustries (Nikel and de Lorenzo, 2018; Weimer et al., 2020). Based on the toolbox for genetic engineering of *P. putida* KT2440 (Poblete-Castro et al., 2020; Sun et al., 2020; Weimer et al., 2020; Zhou et al., 2020), it has been engineered for heterologous production of diverse value-added products (Nikel and de Lorenzo, 2013; Johnson and Beckham, 2015; Dvořák and de Lorenzo, 2018; Sánchez-Pascuala et al., 2019; Bator et al., 2020; Bentley et al., 2020; Tiso et al., 2020) and environmental remediation (Franden et al., 2018). In addition, the intrinsically high metabolic diversity gives *P. putida* KT2440 a wide substrate spectrum for chemical production (Nelson et al., 2002; Wu et al., 2011). Elucidation of the diverse biological processes in *P. putida* KT2440 would incorporate new substrates for bioproduction (Li et al., 2019; Salvachúa et al., 2020; Wada et al., 2021).

Butane-2,3-diol (2,3-BDO) is a physiological metabolic product excreted by many microorganisms such as *Bacillus subtilis*, *B. amyloliquefaciens*, *Klebsiella oxytoca*, *K. pneumoniae*, *Enterobacter cloacae* and *Serratia marcescens* (Ryu et al., 2003; Xu et al., 2014). Some economical 2,3-BDO fermentation processes using various inexpensive carbohydrate raw materials have been established (Liu et al., 2011; Lian et al., 2014; Xu et al., 2014; Chu et al., 2015; Ge et al., 2016; Yang et al., 2017). However, development of efficient derivative processes for 2,3-BDO is still a prerequisite for value-added utilization of biotechnologically produced 2,3-BDO. *P. putida* KT2440 can metabolize 2,3-BDO as the sole carbon source for growth. Recently, a bioconversion process for mevalonate production from 2,3-BDO has been established by using recombinant *P. putida* KT2440 (Yang et al., 2020).

2,3-BDO is a chiral compound with two chiral carbon atoms and it has three stereoisomeric forms, (2*R*,3*R*)-2,3-BDO, *meso*-2,3-BDO, and (2*S*,3*S*)-2,3-BDO. The anabolism mechanism of the three stereoisomeric forms of 2,3-BDO has been detailedly elucidated. Three key enzymes participate in 2,3-BDO biosynthesis. α-Acetolactate synthase catalyzes two molecules of pyruvate into α-acetolactate, which is then converted to (3*R*)-acetoin [(3*R*)-AC] by α-acetolactate decarboxylase (Celińska and Grajek, 2009; Ji et al., 2011). (3*R*)-AC is reduced to *meso*-2,3-BDO and (2*R*,3*R*)-2,3-BDO by *meso*-2,3-BDO dehydrogenase (*meso*-BDH, belonging to short-chain dehydrogenase/reductase SDR family) and (2*R*,3*R*)-2,3-BDO dehydrogenase (*R*,*R*-BDH, belonging to zinc-containing medium-chain dehydrogenase/reductase MDR family), respectively (Celińska and Grajek, 2009; Ji et al., 2011; Xu et al., 2014; Zhang et al., 2014; Yang et al., 2017). In addition, diacetyl, which is produced from α-acetolactate by non-enzymatic oxidative decarboxylation, can be reduced to (2*S*,3*S*)-2,3-BDO by *meso*-BDH (Xiao and Xu., 2007; Ge et al., 2016). Existence of multiple stereospecific dehydrogenases in 2,3-BDO producing bacteria results in the presence of three stereoisomeric forms in natural habitats (Yang et al., 2017). Whether *P. putida* KT2440 can use three stereoisomers of 2,3-BDO has not been clarified.

In this study, *P. putida* KT2440 was identified to be able to use three 2,3-BDO stereoisomers for growth. The first step of 2,3-BDO catabolism is its dehydrogenation to AC and three key enzymes catalyzing different stereoisomers of 2,3-BDO were identified in *P. putida* KT2440. Briefly, *R*,*R*-BDH catalyzes the dehydrogenation of (2*R*,3*R*)-2,3-BDO and *meso*-2,3-BDO. Two quinoprotein alcohol dehydrogenases are critical for (2*S*,3*S*)-2,3-BDO dehydrogenation. In addition, the lanthanide-responsive switch of the two quinoprotein alcohol dehydrogenases was identified to lead to growth of *P. putida* KT2440 in (2*S*,3*S*)-2,3-BDO depending on the presence or absence of lanthanides.

## Materials and Methods

### Bacteria and Culture Conditions

Bacterial strains and plasmids used in this work are listed in Supplementary Table S1. *P. putida* KT2440 and its derivatives were cultured in MSM (Ma et al., 2007) with 2 g L^−1^ different substances at 200 rpm and 30°C. Lanthanide concentration-dependent growth of *P. putida* KT2440 was monitored using a Bioscreen microbiology reader (Bioscreen C Labsystems, Helsinki, Finland). *E. coli* DH5α and BL21 (DE3) were cultured in Luria-Bertani (LB) medium at 180 rpm and 37°C. Antibiotics were added to the medium when necessary, at the following concentrations: kanamycin at 50 μg mL^−1^ and ampicillin at 100 μg mL^−1^.

### Reverse Transcription-Polymerase Chain Reaction

RT-PCR experiments were conducted as described previously (Zhang et al., 2018). Total RNA was isolated from *P. putida* KT2440 cells grown to mid-log phase in MSM supplemented with different carbon sources using Qiagen RNeasy total RNA Kit. DNA contamination was eliminated by RNase-free DNase I treatment. cDNA was generated using Superscript II RT Kit. Samples were incubated at 25°C for 10 min and 42°C for 30 min, then heated at 85°C for 5 min. RT-PCR was performed with Taq DNA Polymerase (Transgen, China) using corresponding oligonucleotides (Supplementary Table S2). The genomic DNA and total RNA of *P. putida* KT2440 were used as positive and negative controls, respectively.

### Expression and Purification of *R*,*R*-BDH

The gene encoding *R*,*R*-BDH was amplified from genomic DNA of *P. putida* KT2440 using primers *pp0552*-F/*pp0552*-R (Supplementary Table S2). The PCR product was cloned into the pETDuet-1 plasmid to obtain plasmid pETDuet-*pp0552*. *E. coli* BL21(DE3) containing pETDuet-*pp0552* was grown to an optical density at 600 nm (OD_600nm_) of 0.6–0.8 in LB medium with 100 μg mL^−1^ ampicillin at 37°C, 180 rpm and induced at 16°C with 1 mM isopropyl-D-1-thiogalactopyranoside (IPTG). The cells were harvested, washed twice, and resuspended in buffer A (pH 7.4, 20 mM sodium phosphate and 500 mM sodium chloride), then lysed by sonication in an ice bath after the addition of 1 mM phenylmethanesulphonyl fluoride (PMSF) and 10% glycerol (vol/vol). The cell lysate was centrifuged at 12,000 × *g* for 40 min at 4°C to remove cell debris. The supernatant was loaded onto a HisTrap HP column (5 mL) and eluted with buffer B (pH 7.4, 20 mM sodium phosphate, 500 mM sodium chloride, and 500 mM imidazole). The quality of purified protein was analyzed by sodium dodecyl sulfate-polyacrylamide gel electrophoresis (SDS-PAGE) with 12.5% polyacrylamide gels and the concentration was determined by the Bradford protein assay Kit.

### Determination of the Native Molecular Weight of *R*,*R*-BDH

The native molecular weight of *R*,*R*-BDH was estimated by using a gel filtration column (Superdex 200 10/300 GL; GE Healthcare, United States) as described previously (Zhang et al., 2019) with the eluent buffer (pH 7.2, 50 mM sodium phosphate and 150 mM sodium chloride) at a flow rate of 0.5 mL min^−1^. Thyroglobulin (669 kDa), ferritin (440 kDa), aldolase (158 kDa), conalbumin (75 kDa), ovalbumin (43 kDa), and ribonuclease A (13.7 kDa) were used as standard proteins.

### Enzymatic Assays of *R*,*R*-BDH

The activity of *R*,*R*-BDH was assayed by measuring the change in absorbance at 340 nm at 30°C. For assay of the oxidative activity, the reaction solution contained 10 mM substrates and 1 mM NAD(P)^+^ in Tris-HCl buffer (pH 7.4, 50 mM). For assay of the reductive activity, the reaction solution contained 5 mM substrates and 0.2 mM NAD(P)H. One unit of enzyme activity was defined as the amount of enzyme that consumed or produced 1 μmol of NAD(P)H per min.

To determine the reductive product of the *R*,*R*-BDH, the 1 ml reaction solution containing 1 mM AC or diacetyl, 1 mM NADH, 10 U purified *R*,*R*-BDH in Tris-HCl (pH 7.4, 50 mM) was incubated at 30°C and 180 rpm for 1 h. The solutions were centrifuged at 13,000 × *g* for 10 min and the supernatant was used for detection. The concentrations of 2,3-BDO and AC were analyzed by a gas chromatography system (Varian 3800, Varian) equipped with a flame ionization detector and a 30-m SPB-5 capillary column (0.32-mm inner diameter, 0.25-μm film thickness; Supelco, Bellefonte, PA) using nitrogen as the carrier gas. The injector and detector temperatures were 280°C while the column oven temperature was maintained at 40°C for 3 min and then raised to 240°C at a rate of 20°C min^−1^ (Ma et al., 2009; Xu et al., 2014). The stereo configuration of 2,3-BDO produced from AC or diacetyl was detected by a gas chromatography system (Agilent GC6820) equipped with a flame ionization detector and a fused silica capillary column (Supelco Beta DEX TM 120, inside diameter, 0.25 mm; length, 30 m) using nitrogen as the carrier gas. The injector and detector temperatures were 280°C while the column oven temperature was maintained at 40°C for 3 min, raised to 80°C at a rate of 1.5°C min^−1^, then increased to 86°C at a rate of 0.5°C min^−1^ and finally raised to 200°C at a rate of 30°C min^−1^ (Xiao et al., 2010).

### Construction of *P. putida* KT2440 Mutants

To construct the Δ*pp0552* mutant strain of *P. putida* KT2440, the upstream and downstream homologous arms of the *pp0552* gene were amplified using the primers *pp0552*-up-F/R and *pp0552*-down-F/R, respectively (Supplementary Table S2). Homologous arms were fused together via recombinant PCR and amplified with the primers *pp0552*-up-F and *pp0552*-down-R. The product was inserted into a mobilizable plasmid pK18*mobsacB* (Schäfer et al., 1994), which does not replicate in *P. putida*, to construct pK18*mobsacB*-Δ*pp0552*. Then the plasmid was transformed into *P. putida* KT2440 by electroporation and integrated into the chromosome. The correct single-crossover mutants were selected on LB plate supplemented with 50 μg mL^−1^ kanamycin. The double-crossover allelic exchange mutant strains were screened on LB plates containing 10% (wt/vol) sucrose. The *pedH* and *pedE* mutants of *P. putida* KT2440 were constructed by using the same procedure.

## Results

### Utilization of Three Stereoisomers of 2,3-BDO by *P. putida* KT2440

**FIGURE 1:**
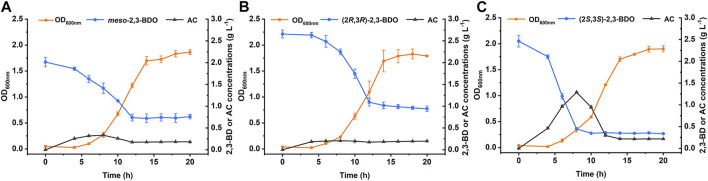
Growth of *P. putida* KT2440 in three stereoisomers of 2,3-BDO. The biomass, 2,3-BDO consumption, and AC production were measured in MSM supplemented with 2 g L^−1^ different stereoisomers of 2,3-BDO as the sole carbon source. **(A)** *meso*-2,3-BDO. **(B)** (2*R*,3*R*)-2,3-BDO. **(C)** (2*S*,3*S*)-2,3-BDO. Error bars indicate deviation from the means (*n* = 3).

Rhizosphere is a natural habitat of different microorganism. Some rhizobacteria such as *Aerobacter* spp., *Bacillus* spp., and *Serratia* spp., can produce three stereoisomers of 2,3-BDO as the volatile compounds to trigger induced systemic resistance and promote plant growth (Ryu et al., 2003; Han et al., 2006; Taghavi et al., 2010). *P. putida* KT2440 is a widely studied rhizosphere-dwelling microorganism. Growth of *P. putida* KT2440 in minimal salt medium (MSM) supplemented with different stereoisomers of 2,3-BDO was firstly studied. As shown in Figure 1, *P. putida* KT2440 grew robustly in all three 2,3-BDO stereoisomers with the consumption of each substrate.

### Comparative Genomics of 2,3-BDO Utilization Operon in *P. putida* KT2440

**FIGURE 2:**
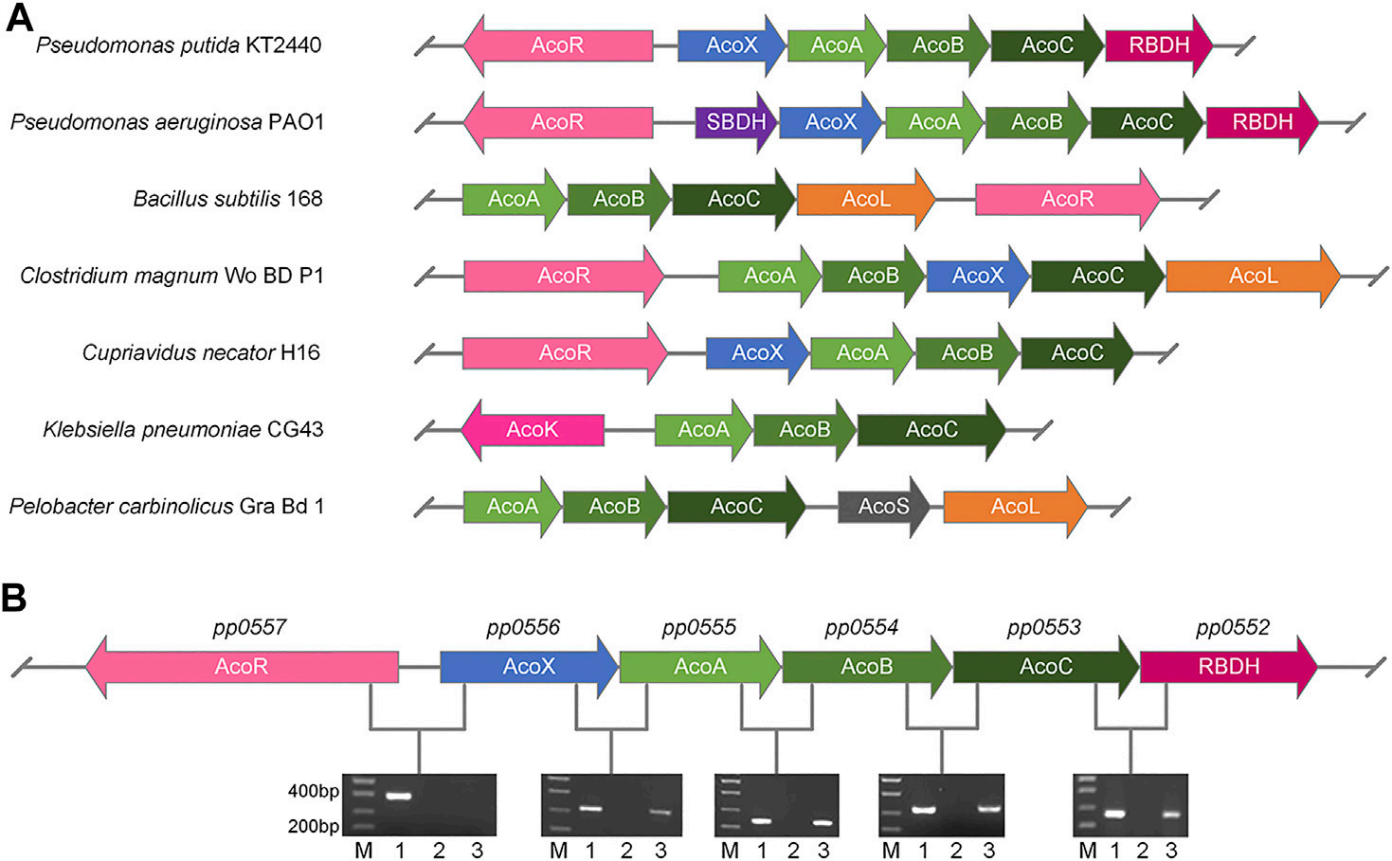
Identification of *R*,*R*-BDH in 2,3-BDO utilization operon of *P. putida* KT2440. **(A)** Gene clusters arrangement of genes for 2,3-BDO and AC catabolism in different species. **(B)** Co-transcription analysis of genes in 2,3-BDO utilization operon of *P. putida* KT2440 by RT-PCR (lane 3). The genomic DNA (lane 1) and RNA (lane 2) of *P. putida* KT2440 were used as positive and negative control, respectively. *P. putida* KT2440 were cultured in MSM supplemented with 2 g L^−1^ mixed 2,3-BDO as the sole carbon source.

Recently, a 2,3-BDO utilization operon was identified in *P. aeruginosa* PAO1 (Liu et al., 2018). Comparative genomics analysis indicated that PP0555, PP0554, and PP0553 exhibit strikingly high homology to AcoA, AcoB, and AcoC in AC dehydrogenase enzyme system (AoDH ES) of *P. aeruginosa* PAO1. *R*,*R*-BDH and (2*S*,3*S*)-2,3-BDO dehydrogenase (*S*,*S*-BDH) encoded in 2,3-BDO utilization operon of *P. aeruginosa* PAO1 catalyze three 2,3-BDO stereoisomers into AC and then AC is cleaved by AoDH ES into acetyl-CoA and acetaldehyde. As shown in Figure 2A, a putative *R*,*R*-BDH (PP0552) is also annotated in the genome of *P. putida* KT2440. Its protein sequence exhibits high homology to *R*,*R*-BDHs of *P. aeruginosa* PAO1 (86.2% sequence identity), *Bacillus cereus* YUF-4 (44.9% sequence identity), *Paenibacillus polymyxa* ATCC 12321 (41.8% sequence identity), *P. polymyxa* ZJ-9 (38.2% sequence identity), *Rhodococcus erythropolis* WZ010 (38.0% sequence identity), and *Saccharomyces cerevisiae* S288C (31.7% sequence identity) **(** Supplementary Figure S1 **)**.

Since *pp0555*, *pp0554*, *pp0553* and *pp0552* were located adjacent to *acoX* (*pp0556*) and *acoR* (*pp0557*) gene homologues of AC utilization operons in other bacteria, we hypothesized that these genes may also comprise a 2,3-BDO utilization operon in *P. putida* KT2440 (Figure 2A). The mRNA from cells of *P. putida* KT2440 grown in 2,3-BDO was extracted and reverse-transcribed into cDNA as template for reverse transcription-polymerase chain reaction (RT-PCR). As shown in Figure 2B, the five genes including *pp0556*, *pp0555*, *pp0554*, *pp0553* and *pp0552* were co-transcribed and might be regulated by AcoR.

### Enzymatic Properties of *R*,*R*-BDH in *P. putida* KT2440

**FIGURE 3:**
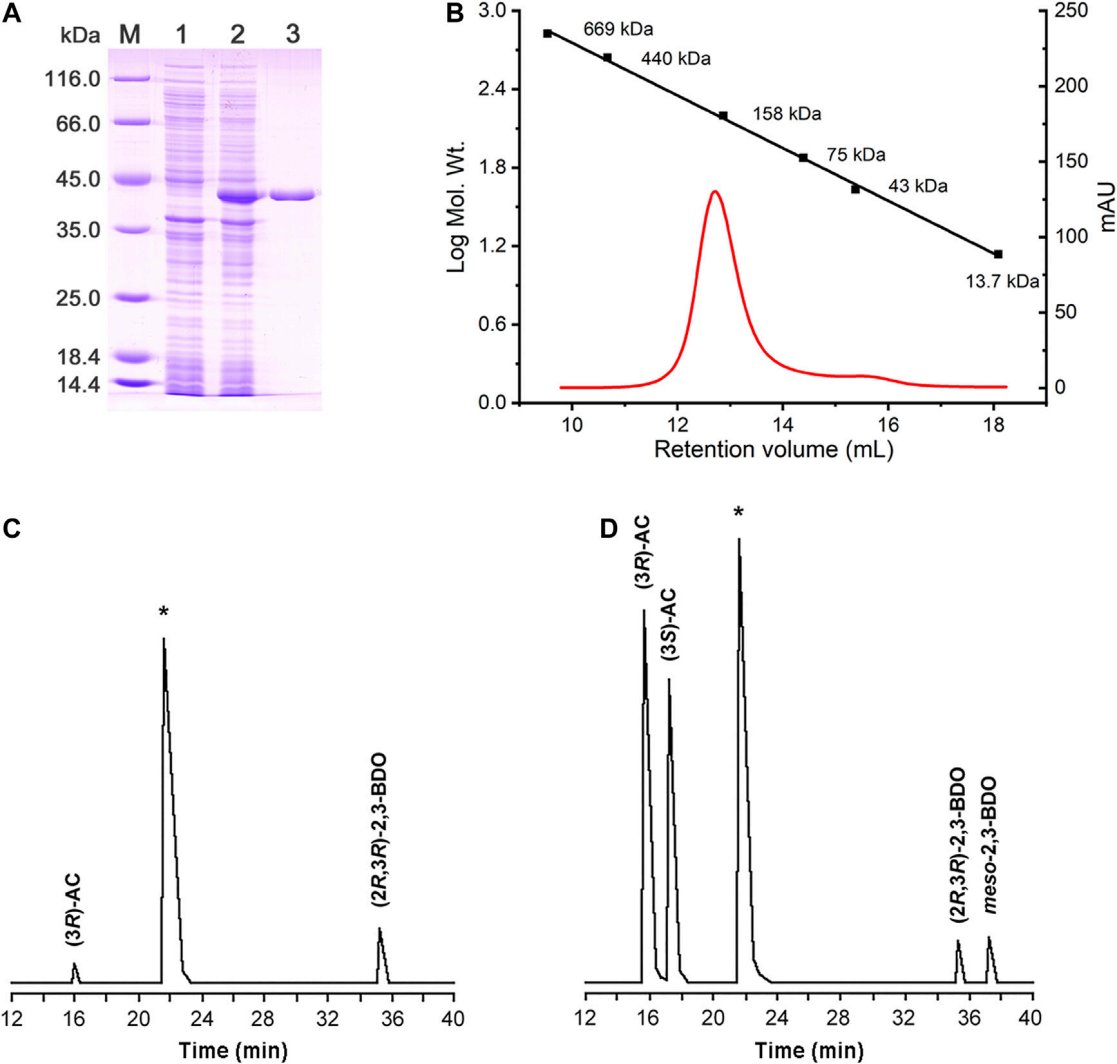
Enzymatic characterization of *R*,*R*-BDH in *P. putida* KT2440. **(A)** SDS-PAGE analysis of the purified *R*,*R*-BDH. Lane M, molecular weight markers; Lane 1, crude cell extract of *E. coli* BL21 (DE3) (blank control); Lane 2, crude cell extract of *E. coli* BL21 (DE3) harboring pETDuet-*pp0552*; Lane 3, purified *R*,*R*-BDH. **(B)** Determination of the native molecular weight of *R*,*R*-BDH by gel filtration column. GC analysis of 2,3-BDO produced by *R*,*R*-BDH catalyzed reduction of diacetyl **(C)** and racemic AC **(D)**.

The putative *R*,*R*-BDH in *P. putida* KT2440 was overexpressed and then purified by using a HisTrap column (Figure 3A). Based on the mass of the *R*,*R*-BDH monomer and the results of size exclusion chromatography, this protein exists as a homotetramer (Figure 3B). For the oxidative reaction, *R*,*R*-BDH can catalyze NAD-dependent dehydrogenation of (2*R*,3*R*)-2,3-BDO and *meso*-2,3-BDO. For the reductive reaction, *R*,*R*-BDH can catalyze NADH-dependent reduction of diacetyl and AC (Table 1 and Table 2). It had no activity toward 1,3-butanediol, 2-butanol, 1,3-propanediol, and glycerol.

**TABLE 1:** The relative activity of *R*,*R*-BDH toward different substrates.

Substrate	Relative activity (%)^a^
Oxidative reaction^b^	
*meso*-2,3-BDO	100
(2*R*,3*R*)-2,3-BDO	61.4
(2*S*,3*S*)-2,3-BDO	ND^d^
1,3-BDO	ND
1,2-BDO	44.6
2-Butanol	ND
1,2-Propanediol	22.9
1,3-Propanediol	ND
Glycerol	ND
Reductive reaction^c^	
Acetoin	100
Diacetyl	74.7
2,3-Pentanedione	77.8
2,4-Pentanedione	ND
n-Butanal	ND

a The highest specific activity of oxidation or reduction was defined as 100%.

b Oxidative activity was examined with 10 mM substrates and 1 mM  NAD^+^ in 50 mM Tris-HCl buffer (pH 7.4) at 30°C.

c Reductive activity was examined with 5 mM substrates and 0.2 mM NADH in 50 mM Tris-HCl buffer (pH 7.4) at 30°C.

d ND means not detected.

**TABLE 2:** Identification of the cofactor of *R*,*R*-BDH.

Substrate	Specific activity (U^a^ mg^−1^)	
	NAD^+^(NADH)	NADP^+^(NADPH)
Acetoin	9.015 ± 0.165	ND^b^
*meso*-2,3-BDO	4.004 ± 0.288	ND
(2*R*,3*R*)-2,3-BDO	3.572 ± 0.270	ND
(2*S*,3*S*)-2,3-BDO	ND	ND

a One unit of *R*,*R*-BDH activity was defined as the amount of enzyme that consumed or produced 1 μmol of NAD(P)H per min. Data shown are mean ± s.d. (*n* = 3 independent experiments).

b ND means not detected.

The kinetic parameters of *R*,*R*-BDH in *P. putida* KT2440 toward different substrates were determined (Table 3). In oxidative direction, double-reciprocal plots of the initial rates plotted against the concentrations of (2*R*,3*R*)-2,3-BDO, *meso*-2,3-BDO, and cofactor NAD^+^ yielded *K*_m_ values of 0.155 ± 0.016 mM, 0.251 ± 0.022, and 0.825 ± 0.096 mM, respectively. The *k*_cat_ values were estimated to be 23.858 ± 1.300 s^−1^, 21.065 ± 0.022 s^−1^, and 52.821 ± 3.798 s^−1^, respectively. *R*,*R*-BDH in *P. putida* KT2440 exhibited higher catalytic efficiency toward *meso*-2,3-BDO and (2*R*,3*R*)-2,3-BDO dehydrogenation than other reported *R*,*R*-BDHs (Supplementary Table S3). In reductive direction, the apparent *K*_m_ values of *R*,*R*-BDH toward AC and NADH was 0.101 ± 0.005 mM and 0.119 ± 0.022 mM, respectively. The *k*_cat_ values were estimated to be 34.336 ± 0.256 s^−1^ and 53.296 ± 5.164 s^−1^, respectively.

**TABLE 3:** Kinetic parameters of *R*,*R*-BDH.

Substrate	*k*_cat_ (s^−1^)	*K*_m_ (mM)	*k*_cat_/*K*_m_ (s^−1^·mM^−1^)
*meso*-2,3-BDO	21.065 ± 0.022	0.251 ± 0.022	84.328 ± 5.799
(2*R*,3*R*)-2,3-BDO	23.858 ± 1.300	0.155 ± 0.016	154.186 ± 9.227
Acetoin	34.336 ± 0.256	0.101 ± 0.005	341.387 ± 16.864
NAD^+^	52.821 ± 3.798	0.825 ± 0.096	64.285 ± 3.115
NADH	53.296 ± 5.164	0.119 ± 0.022	453.857 ± 36.564

Data shown are mean ± s.d. (*n* = 3 independent experiments).

The effects of pH and temperature on the enzyme activity and stability of *R*,*R*-BDH were examined. The optimal pH for oxidative activity of *R*,*R*-BDH toward (2*R*,3*R*)-2,3-BDO **(** Supplementary Figure S2A **)** and *meso*-2,3-BDO (Supplementary Figure S2B) is 9.0, while for reductive activity toward AC is 8.0 (Supplementary Figure S2C). The enzyme is more stable under the pH range of 6.0–8.0 in citrate-Na_2_HPO_4_ buffer (Supplementary Figure S2D). The optimum temperature for *R*,*R*-BDH is about 70°C (Supplementary Figure S3A) and it is stable under 50°C (Supplementary Figure S3B). The effect of metal ions on the activity of *R*,*R*-BDH was tested by adding different metal salts (final concentration of 1 mM) to the assay buffer. As shown in Supplementary Figure S4, Fe^2+^, Mn^2+^, Co^2+^, Ca^2+^, Ba^2+^, Mg^2+^, and K^+^ have positively effects on *R*,*R*-BDH activity, while Zn^2+^, Cd^2+^, Cu^2+^, Ni^2+^, and EDTA have negative effects.

2,3-BDHs catalyze the stereoselective interconversion between diacetyl, 2,3-BDO and AC. As shown in Figure 3C, diacetyl was reduced into (3*R*)-AC and (2*R*,3*R*)-2,3-BDO by *R*,*R*-BDH, whereas racemic AC was reduced to (2*R*,3*R*)-2,3-BDO and *meso*-2,3-BDO (Figure 3D). These results indicated that *R*,*R*-BDH catalyzes the *R*-specific oxidation-reduction of hydroxyl and keto groups in its substrates and (2*R*,3*R*)-2,3-BDO and *meso*-2,3-BDO might be converted to (3*R*)-AC and (3*S*)-AC, respectively.

### Functions of *R*,*R*-BDH in (2*R*,3*R*)-2,3-BDO and *meso*-2,3-BDO Utilization of *P. putida* KT2440

**FIGURE 4:**
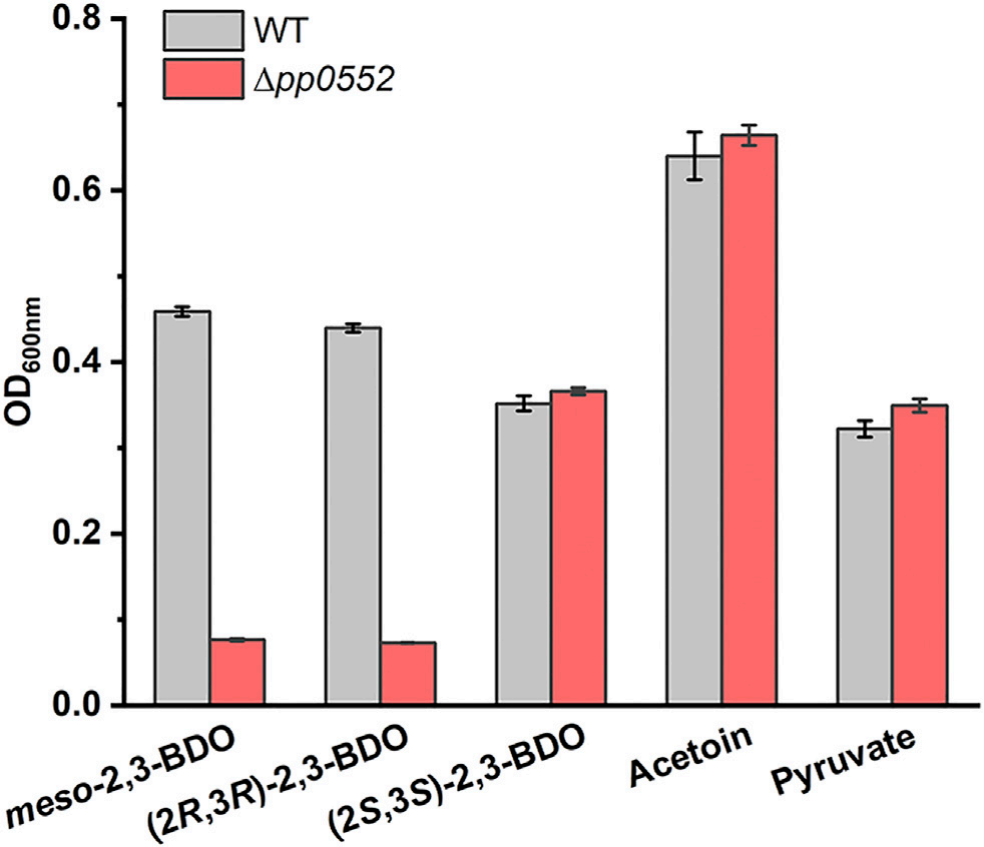
Identification of the physiological function of *R*,*R*-BDH in 2,3-BDO catabolism. *P. putida* KT2440 and *P. putida* KT2440 (Δ*pp0552*) were cultured in MSM with 2 g L^−1^ *meso*-2,3-BDO, (2*R*,3*R*)-2,3-BDO, (2*S*,3*S*)-2,3-BDO, racemic AC, or pyruvate as the sole carbon source at 30°C for 30 h by using a Bioscreen microbiology reader. Error bars indicate deviation from the means (*n* = 3).

RT-PCR fragment of *pp0552* was obtained from cells of *P. putida* KT2400 cultured with (2*R*,3*R*)-2,3-BDO, *meso*-2,3-BDO and (2*S*,3*S*)-2,3-BDO (Supplementary Figure S5A), indicating the transcription of *pp0552* during 2,3-BDO utilization. To identify the role of *R*,*R*-BDH in 2,3-BDO utilization, its encoding gene *pp0552* was knocked out in *P. putida* KT2440. *P. putida* KT2440 (Δ*pp0552*) was cultivated in MSM supplemented with 2 g L^−1^ *meso*-2,3-BDO, (2*R*,3*R*)-2,3-BDO, (2*S*,3*S*)-2,3-BDO, racemic AC or pyruvate. As shown in Figure 4 **,** the disruption of *pp0552* had no effect on the ability of *P. putida* KT2440 (Δ*pp0552*) to grow in racemic AC and pyruvate but impaired its ability to utilize *meso*-2,3-BDO and (2*R*,3*R*)-2,3-BDO. This result indicated that *R*,*R*-BDH plays indispensable roles in *meso*-2,3-BDO and (2*R*,3*R*)-2,3-BDO catabolism, definitely through converting these two stereoisomers into AC. *P. putida* KT2440 (Δ*pp0552*) could still utilize (2*S*,3*S*)-2,3-BDO, suggesting the presence of other proteins responding for (2*S*,3*S*)-2,3-BDO catabolism.

### Quinoprotein Ethanol Dehydrogenases Involved in (2*S*,3*S*)-2,3-BDO Utilization of *P. putida* KT2440

**FIGURE 5:**
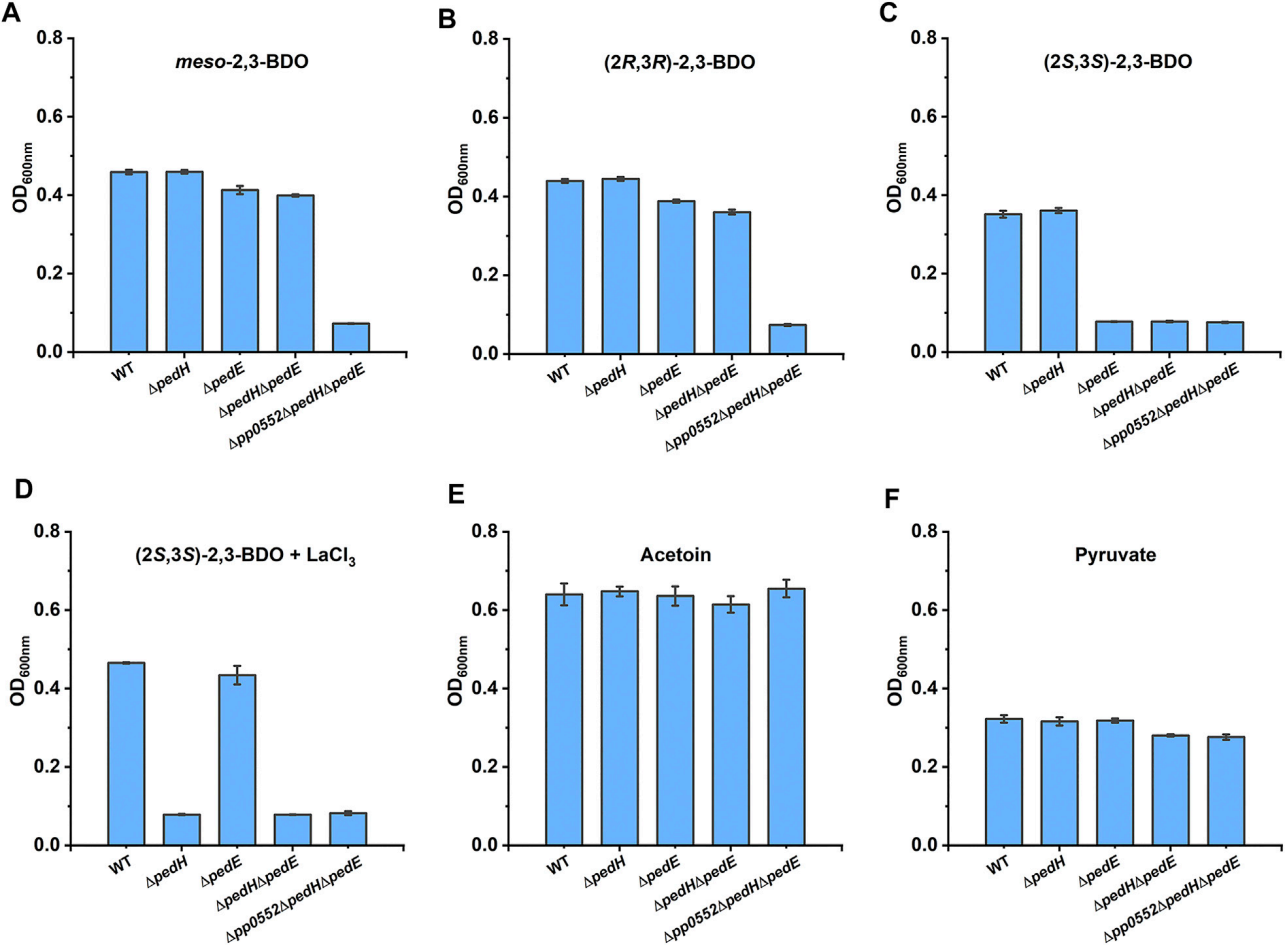
Identification of the physiological function of PedE and PedH in 2,3-BDO catabolism. Individual and combinatorial deletion mutants of *P. putida* KT2440 were cultured in MSM supplemented with 2 g L^−1^ *meso*-2,3-BDO **(A)**, (2*R*,3*R*)-2,3-BDO **(B)**, (2*S*,3*S*)-2,3-BDO **(C)**, (2*S*,3*S*)-2,3-BDO with 20 μM LaCl_3_ **(D)**, racemic AC **(E)** and pyruvate **(F)** as the sole carbon source at 30°C for 30 h by using a Bioscreen microbiology reader. Error bars indicate deviation from the means (*n* = 3).

Two pyrroloquinoline quinone (PQQ)-dependent ethanol dehydrogenases (PQQ-EDHs), PP2674 (PedE) and PP2679 (PedH), were annotated in the genome of *P. putida* KT2440. PedH and PedE exhibit enzyme activity toward a range of substrates, including 2,3-BDO (Takeda et al., 2013; Wehrmann et al., 2017). In addition, transcription of *pedE* and *pedH* was also observed during (2*R*,3*R*)-2,3-BDO, *meso*-2,3-BDO and (2*S*,3*S*)-2,3-BDO utilization of *P. putida* KT2440 (Supplementary Figure S5B and Supplementary Figure S5C). This makes us wonder whether these two PQQ-EDHs also participate in 2,3-BDO catabolism of *P. putida* KT2440. Individual or combinatorial mutant strains of PedE, PedH, and *R*,*R*-BDH were constructed. As shown in Figure 5A and Figure 5B, knock out of *pedE* and/or *pedH* did not impair the growth of *P. putida* KT2440 in *meso*-2,3-BDO and (2*R*,3*R*)-2,3-BDO. *P. putida* KT2440 (Δ*pedE*) displayed no growth in MSM with (2*S*,3*S*)-2,3-BDO as sole carbon source, while *P. putida* KT2440 (Δ*pedH*) still grew normally under the same condition (Figure 5C).

PedE depends on Ca^2+^ as a cofactor (Takeda et al., 2013), while PedH exhibits enzyme activity only in the presence of La^3+^ (Wehrmann et al., 2017). Thus, 20 μM La^3+^ was added in MSM with (2*S*,3*S*)-2,3-BDO as the sole carbon source, which resulted in robust growth of *P. putida* KT2440 (Δ*pedE*) (Figure 5D). Double-knockout strain *P. putida* KT2440 (Δ*pedE*Δ*pedH*) was completely unable to utilize (2*S*,3*S*)-2,3-BDO both in the presence and absence of La^3+^ (Figures 5C,D) and there might be no other alcohol dehydrogenase catalyzing dehydrogenation of (2*S*,3*S*)-2,3-BDO in the strain. PedE and PedH function redundantly in (2*S*,3*S*)-2,3-BDO catabolism, but work under different conditions. The triple-knockout strain *P. putida* KT2440 (Δ*pp0552*Δ*pedE*Δ*pedH*) grew normally in AC (Figure 5E) and pyruvate (Figure 5F), while lost growth ability in either stereoisomer of 2,3-BDO (Figures 5A–D). Thus, *P. putida* KT2440 uses *R*,*R*-BDH, PedH, and PedE to utilize different 2,3-BDO stereoisomers.

### Lanthanide Concentration-dependent Growth of Derivative Strains of *P. putida* KT2440 in (2*S,*3*S*)-2,3-BDO

The transcription of *pedE* and *pedH* is tightly controlled by a lanthanide-mediated switch in *P. putida* KT2440 (Wehrmann et al., 2017; Wehrmann et al., 2018). In the presence of La^3+^, *pedE* transcription is activated while *pedH* is repressed. Thus, the expression of PedH and PedE will be inversely regulated in response to various lanthanide concentrations and the lanthanide-mediated switch also influences the PQQ-EDH dependent growth of *P. putida* KT2440. Growth of derivative strains of *P. putida* KT2440 in (2*S*,3*S*)-2,3-BDO was assayed in the presence of various concentration of La^3+^.

**FIGURE 6:**
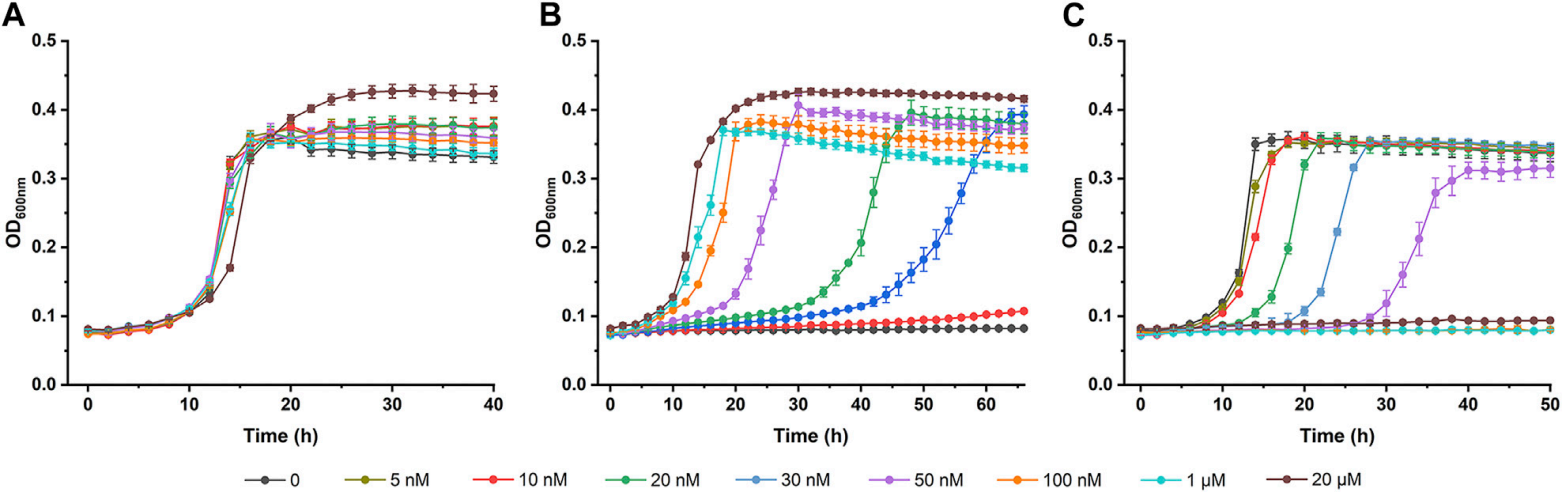
Lanthanide concentration-dependent growth of derivative strains of *P. putida* KT2440 in (2*S*,3*S*)-2,3-BDO. *P. putida* KT2440 wild-type **(A)**, Δ*pedE* **(B)** and Δ*pedH* **(C)** mutant strains were cultured in MSM supplemented with 2 g L^−1^ (2*S*,3*S*)-2,3-BDO with various concentrations of LaCl_3_ at 30°C by using a Bioscreen microbiology reader. Error bars indicate deviation from the means (*n* = 3).

As shown in Figure 6B, the growth of *P. putida* KT2440 (Δ*pedE*) in (2*S*,3*S*)-2,3-BDO was activated by increasing La^3+^ concentrations. In contrast, La^3+^ concentrations-dependent growth inhibition of *P. putida* KT2440 (Δ*pedH*) was also detected (Figure 6C). *P. putida* KT2440 (Δ*pedH*) did not grow in (2*S*,3*S*)-2,3-BDO when La^3+^ concentration was higher than 100 nM. Importantly, the growth of wild type *P. putida* KT2440 was not affected whether exogenous La^3+^ was existent or not (Figure 6A). *P. putida* KT2440 may be conferred a strong survivability by the redundancy of PedH and PedE expressed under different conditions.

## Discussion

**FIGURE 7:**
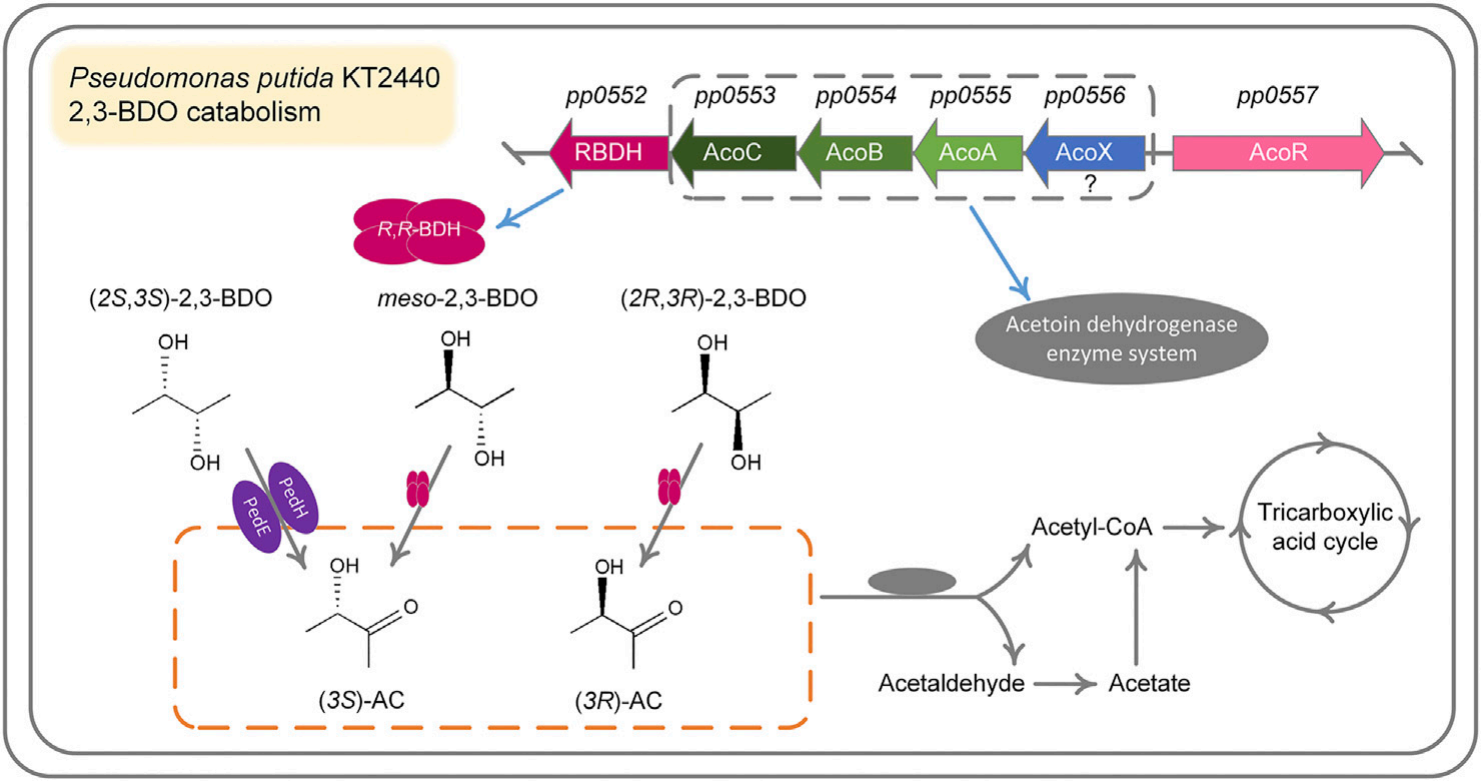
Proposed mechanism for 2,3-BDO catabolism in *P. putida* KT2440. *meso*-2,3-BDO and (2*R*,3*R*)-2,3-BDO are dehydrogenized by *R*,*R*-BDH to (3*S*)-AC and (3*R*)-AC, respectively. Dehydrogenation of (2*S*, 3*S*)-2,3-BDO to (3*S*)-AC is catalyzed by two PQQ-EDHs, PedH and PedE encoded by *pp2679* and *pp2674*, respectively. (3*S*)-AC and (3*R*)-AC are subsequently cleaved by AoDH ES to acetyl-CoA and acetaldehyde for further metabolism.

2,3-BDO is widely distributed in various environments (Ryu et al., 2003; Yi et al., 2016). As a Voges-Proskauer reaction negative strain lacking the ability to produce 2,3-BDO, *P. putida* KT2440 can use all three stereoisomers of 2,3-BDO for its growth. The ability to efficiently use three stereoisomers of 2,3-BDO may be important for *P. putida* to survive and acquire nutrients in habitats like rhizosphere. Dehydrogenation to AC is the first step of 2,3-BDO catabolism and then AC will be cleaved into acetaldehyde and acetyl-CoA by AoDH ES (Xiao and Xu., 2007; Liu et al., 2018). In this work, we identified the dehydrogenation mechanism of three stereoisomers of 2,3-BDO: (2*R*,3*R*)-2,3-BDO, *meso*-2,3-BDO, and (2*S*,3*S*)-2,3-BDO in *P. putida* KT2440 (Figure 7). *R*,*R*-BDH catalyzes the dehydrogenation of (2*R*,3*R*)-2,3-BDO and *meso*-2,3-BDO with nearly identical *k*_cat_ values, 23.858 ± 1.300 s^−1^ and 21.065 ± 0.022 s^−1^. Similar growth and consumption of substate were observed when *P. putida* KT2440 was cultured with (2*R*,3*R*)-2,3-BDO and *meso*-2,3-BDO (Figures 1A,B). PedE and PedH catalyze the irreversible dehydrogenation of (2*S*,3*S*)-2,3-BDO into (3*S*)-AC. Quickly consumption of (2*S*,3*S*)-2,3-BDO and temporary accumulation of AC can be detected during the (2*S*,3*S*)-2,3-BDO utilization of *P. putida* KT2440 (Figure 1C).

BDHs in Voges-Proskauer reaction positive strains can be divided into MDR family and SDR family (Radoš et al., 2016; Liu et al., 2018). *R*,*R*-BDH belonging to MDR family is critical for (2*R*,3*R*)-2,3-BDO and *meso*-2,3-BDO generation and reutilization, while *meso*-BDH belonging to the SDR family is responsible for (2*S*,3*S*)-2,3-BDO and *meso*-2,3-BDO production and reutilization. The genes encoding *R*,*R*-BDH and *S*,*S*-BDH, which is only active on (2*S*,3*S*)-2,3-BDO and specifically involved in (2*S*,3*S*)-2,3-BDO metabolism, are also present in Voges-Proskauer reaction negative *P. aeruginosa* PAO1 and belong to an operon (Liu et al., 2018). Although there is no *meso*-BDH and *S*,*S*-BDH encoding genes in *P. putida* KT2440, this strain can still catabolize (2*S*,3*S*)-2,3-BDO through two PQQ-EDHs, PedE and PedH.

PedE and PedH exhibited enzyme activity toward many linear and aromatic primary and secondary alcohols, including 2,3-BDO (Takeda et al., 2013; Wehrmann et al., 2017). It was recently reported that mutant strains with either single-knockout or double-knockout of PedE and PedH could still grow on the plate with 2,3-BDO as sole carbon source (Wehrmann et al., 2017). This phenomenon is presumably because that mixed 2,3-BDO of three stereoisomers was used to culture those mutant strains. Although the catabolism of (2*S*,3*S*)-2,3-BDO was abolished due to the deletion of PedE and PedH, *R*,*R*-BDH could still catalyze the dehydrogenation of (2*R*,3*R*)-2,3-BDO and *meso*-2,3-BDO in the mixture and support the growth. In addition, we demonstrated that PedE and PedH are specifically involved in (2*S*,3*S*)-2,3-BDO metabolism. In spite of the presence of PedE and PedH, *P. putida* KT2440 (Δ*pp0552*) could not grow in *meso*-2,3-BDO and (2*R*,3*R*)-2,3-BDO. Thus, when using 2,3-BDO as the substrate, PQQ-EDHs may be stereospecific and only active on (2*S*,3*S*)-2,3-BDO.

The growth of *P. putida* KT2440 (Δ*pedE*) in (2*S*,3*S*)-2,3-BDO was exclusively stimulated by increased concentrations of lanthanum. In contrast, the growth of *P. putida* KT2440 (Δ*pedH*) in (2*S*,3*S*)-2,3-BDO was inhibited by lanthanum in a concentration-dependent manner. These results were due to the inverse regulation of PedE and PedH by lanthanide availability, an interesting phenomenon called as lanthanide-mediated switch in several methylotrophic organisms and *P. putida* KT2440 (Farhan Ul Haque et al., 2015; Chu et al., 2016; Vu et al., 2016; Wehrmann et al., 2017; Wehrmann et al., 2018). Since low concentrations of lanthanum could still inhibit the growth of *P. putida* KT2440 (Δ*pedH*) in (2*S*,3*S*)-2,3-BDO at the presence of Ca^2+^, the lanthanide-dependent PedH seems to be the preferred PQQ-EDH when both metals are simultaneously available (Chu and Lidstrom, 2016; Vu et al., 2016; Wehrmann et al., 2017). In addition, the functional redundancy and inverse regulation of PQQ-EDHs provides a mechanism through which *P. putida* KT2440 can optimize its growth according to the availability of lanthanide under variable environmental conditions (Markert, 1987; Aubert et al., 2002).

*P. putida* KT2440 can colonize rhizospheres of different plants and induce changes in root exudation of plants like *Arabidopsis* (Matilla et al., 2010). Interestingly, plant root exudates also regulate expression of genes for 2,3-BDO catabolism and synthesis of some antifungal compounds in rhizosphere *Pseudomonas* (Mavrodi et al., 2021). As an extracellular metabolite, 2,3-BDO can act as a signaling molecule influencing the action of various organisms in symbiotic habitat of 2,3-BDO producing strains. For example, 2,3-BDO can be a kairomone or pheromone attracting insects and influencing insect propagation (Rochat et al., 2000; Moore et al., 2002). *P. putida* KT2440 can use endogenous chemical signals to coordinate the expression of various genes (Espinosa-Urgel and Ramos, 2004). Whether 2,3-BDO acts as a signaling molecule of *P. putida* KT2440 like the situation in insects needs further research. In addition, diverse mechanisms for dehydrogenation of three 2,3-BDO stereoisomers in *P. putida* KT2440 were ascertained in this work, whether and how the three isomers exhibit different signaling functions also deserve intensive investigation.

In summary, we proved here that *P. putida* KT2440 is able to utilize all three stereoisomers of 2,3-BDO for growth and revealed the dehydrogenation mechanisms of these stereoisomers. Catabolism of 2,3-BDO generates two molecules of acetyl-CoA without carbon loss (Yang et al., 2020) and thus 2,3-BDO is an ideal substrate for the production of chemicals using acetyl-CoA as the precursor. Chemical production of 2,3-BDO based on nonrenewable resources often results in a mixture of three stereoisomeric forms. Many microorganisms have been used to efficiently produce 2,3-BDO but most of them also produce a mixture of two or three stereoisomers of 2,3-BDO. The results reported in this work thus also provide a molecular basis for efficient utilization of chemical or biological produced 2,3-BDO by *P. putida* KT2440.

## Data Availability

The original contributions presented in the study are included in the article/Supplementary Material, further inquiries can be directed to the corresponding author.

## References

[B1] Aubert D. Stille P. Probst A. Gauthier-lafaye F. Pourcelot L. Del Nero M. (2002). Characterization and Migration of Atmospheric REE in Soils and Surface Waters. Geochim. Cosmochim. Acta 66, 3339–3350. 10.1016/S0016-7037(02)00913-4

[B2] Bator I. Wittgens A. Rosenau F. Tiso T. Blank L. M. (2020). Comparison of Three Xylose Pathways in *Pseudomonas putida* KT2440 for the Synthesis of Valuable Products. Front. Bioeng. Biotechnol. 7, 480. 10.3389/fbioe.2019.00480 32010683 PMC6978631

[B3] Bentley G. J. Narayanan N. Jha R. K. Salvachúa D. Elmore J. R. Peabody G. L. (2020). Engineering Glucose Metabolism for Enhanced Muconic Acid Production in *Pseudomonas putida* KT2440. Metab. Eng. 59, 64–75. 10.1016/j.ymben.2020.01.001 31931111

[B4] Celińska E. Grajek W. (2009). Biotechnological Production of 2,3-Butanediol-Current State and Prospects. Biotechnol. Adv. 27, 715–725. 10.1016/j.biotechadv.2009.05.002 19442714

[B5] Chu F. Beck D. A. C. Lidstrom M. E. (2016). MxaY Regulates the Lanthanide-Mediated Methanol Dehydrogenase Switch in *Methylomicrobium Buryatense* . PeerJ 4, e2435. 10.7717/peerj.2435 27651996 PMC5018670

[B6] Chu F. Lidstrom M. E. (2016). XoxF Acts as the Predominant Methanol Dehydrogenase in the Type I Methanotroph *Methylomicrobium buryatense* . J. Bacteriol. 198, 1317–1325. 10.1128/JB.00959-15 26858104 PMC4859581

[B7] Chu H. Xin B. Liu P. Wang Y. Li L. Liu X. (2015). Metabolic Engineering of *Escherichia coli* for Production of (2*S*,3*S*)-Butane-2,3-Diol from Glucose. Biotechnol. Biofuels 8, 143. 10.1186/s13068-015-0324-x 26379775 PMC4570510

[B8] Dvořák P. de Lorenzo V. (2018). Refactoring the Upper Sugar Metabolism of *Pseudomonas putida* for Co-utilization of Cellobiose, Xylose, and Glucose. Metab. Eng. 48, 94–108. 10.1016/j.ymben.2018.05.019 29864584

[B9] Espinosa-Urgel M. Ramos J. L. (2004). Cell Density-dependent Gene Contributes to Efficient Seed Colonization by *Pseudomonas putida* KT2440. Appl. Environ. Microbiol. 70, 5190–5198. 10.1128/AEM.70.9.5190-5198.2004 15345399 PMC520864

[B10] Farhan Ul Haque M. Kalidass B. Bandow N. Turpin E. A. DiSpirito A. A. Semrau J. D. (2015). Cerium Regulates Expression of Alternative Methanol Dehydrogenases in *Methylosinus trichosporium* OB3b. Appl. Environ. Microbiol. 81, 7546–7552. 10.1128/AEM.02542-15 26296730 PMC4592857

[B11] Franden M. A. Jayakody L. N. Li W. J. Wagner N. J. Cleveland N. S. Michener W. E. (2018). Engineering *Pseudomonas putida* KT2440 for Efficient Ethylene Glycol Utilization. Metab. Eng. 48, 197–207. 10.1016/j.ymben.2018.06.003 29885475

[B12] Ge Y. Li K. Li L. Gao C. Zhang L. Ma C. (2016). Contracted but Effective: Production of Enantiopure 2,3-Butanediol by Thermophilic and GRAS *Bacillus licheniformis* . Green Chem. 18, 4693–4703. 10.1039/c6gc01023g

[B13] Han S. H. Lee S. J. Moon J. H. Park K. H. Yang K. Y. Cho B. H. (2006). GacS-dependent Production of 2R, 3R-Butanediol by *Pseudomonas chlororaphis* O6 Is a Major Determinant for Eliciting Systemic Resistance against *Erwinia carotovora* but Not against *Pseudomonas syringae* pv. *Tabaci* in Tobacco. Mol. Plant Microbe Interact. 19, 924–930. 10.1094/MPMI-19-0924 16903358

[B14] Ji X. J. Huang H. Ouyang P. K. (2011). Microbial 2,3-Butanediol Production: A State-of-the-Art Review. Biotechnol. Adv. 29, 351–364. 10.1016/j.biotechadv.2011.01.007 21272631

[B15] Johnson C. W. Beckham G. T. (2015). Aromatic Catabolic Pathway Selection for Optimal Production of Pyruvate and Lactate from Lignin. Metab. Eng. 28, 240–247. 10.1016/j.ymben.2015.01.005 25617773

[B16] Li W. J. Jayakody L. N. Franden M. A. Wehrmann M. Daun T. Hauer B. (2019). Laboratory Evolution Reveals the Metabolic and Regulatory Basis of Ethylene Glycol Metabolism by *Pseudomonas putida* KT2440. Environ. Microbiol. 21, 3669–3682. 10.1111/1462-2920.14703 31166064

[B17] Lian J. Chao R. Zhao H. (2014). Metabolic Engineering of a *Saccharomyces cerevisiae* Strain Capable of Simultaneously Utilizing Glucose and Galactose to Produce Enantiopure (2*R*,3*R*)-Butanediol. Metab. Eng. 23, 92–99. 10.1016/j.ymben.2014.02.003 24525332

[B18] Liu Q. Liu Y. Kang Z. Xiao D. Gao C. Xu P. (2018). 2,3-Butanediol Catabolism in *Pseudomonas aeruginosa* PAO1. Environ. Microbiol. 20, 3927–3940. 10.1111/1462-2920.14332 30058099

[B19] Liu Z. Qin J. Gao C. Hua D. Ma C. Li L. (2011). Production of (2*S*,3*S*)-2,3-Butanediol and (3*S*)-Acetoin from Glucose Using Resting Cells of *Klebsiella pneumonia* and *Bacillus subtilis* . Bioresour. Technol. 102, 10741–10744. 10.1016/j.biortech.2011.08.110 21945208

[B20] Ma C. Gao C. Qiu J. Hao J. Liu W. Wang A. (2007). Membrane-bound L- and D-Lactate Dehydrogenase Activities of a Newly Isolated *Pseudomonas stutzeri* Strain. Appl. Microbiol. Biotechnol. 77, 91–98. 10.1007/s00253-007-1132-4 17805529

[B21] Ma C. Wang A. Qin J. Li L. Ai X. Jiang T. (2009). Enhanced 2,3-Butanediol Production by *Klebsiella pneumoniae* SDM. Appl. Microbiol. Biotechnol. 82, 49–57. 10.1007/s00253-008-1732-7 18949476

[B22] Markert B. (1987). The Pattern of Distribution of Lanthanide Elements in Soils and Plants. Phytochemistry 26, 3167–3170. 10.1016/S0031-9422(00)82463-2

[B23] Matilla M. A. Ramos J. L. Bakker P. A. H. M. Doornbos R. Badri D. V. Vivanco J. M. (2010). *Pseudomonas putida* KT2440 Causes Induced Systemic Resistance and Changes in *Arabidopsis* Root Exudation. Environ. Microbiol. Rep. 2, 381–388. 10.1111/j.1758-2229.2009.00091.x 23766110

[B24] Mavrodi O. V. McWilliams J. R. Peter J. O. Berim A. Hassan K. A. Elbourne L. D. H. (2021). Root Exudates Alter the Expression of Diverse Metabolic, Transport, Regulatory, and Stress Response Genes in Rhizosphere *Pseudomonas* . Front. Microbiol. 12, 651282. 10.3389/fmicb.2021.651282 33936009 PMC8079746

[B25] Moore A. J. Haynes K. F. Preziosi R. F. Moore P. J. (2002). The Evolution of Interacting Phenotypes: Genetics and Evolution of Social Dominance. Am. Nat. 160, S186–S197. 10.1086/342899 18707476

[B26] Nelson K. E. (2002). The Complete Genome Sequence of *Pseudomonas putida* KT2440 Is Finally Available. Environ. Microbiol. 4, 777–778. 10.1046/j.1462-2920.2002.00367.x

[B27] Nelson K. E. Weinel C. Paulsen I. T. Dodson R. J. Hilbert H. Martins dos Santos V. A. P. (2002). Complete Genome Sequence and Comparative Analysis of the Metabolically Versatile *Pseudomonas putida* KT2440. Environ. Microbiol. 4, 799–808. 10.1046/j.1462-2920.2002.00366.x 12534463

[B28] Nikel P. I. de Lorenzo V. (2013). Engineering an Anaerobic Metabolic Regime in *Pseudomonas putida* KT2440 for the Anoxic Biodegradation of 1,3-Dichloroprop-1-Ene. Metab. Eng. 15, 98–112. 10.1016/j.ymben.2012.09.006 23149123

[B29] Nikel P. I. de Lorenzo V. (2018). *Pseudomonas putida* as a Functional Chassis for Industrial Biocatalysis: from Native Biochemistry to Trans-metabolism. Metab. Eng. 50, 142–155. 10.1016/j.ymben.2018.05.005 29758287

[B30] Poblete-Castro I. Aravena-Carrasco C. Orellana-Saez M. Pacheco N. Cabrera A. Borrero-de Acuña J. M. (2020). Engineering the Osmotic State of *Pseudomonas putida* KT2440 for Efficient Cell Disruption and Downstream Processing of Poly(3-Hydroxyalkanoates). Front. Bioeng. Biotechnol. 8, 161. 10.3389/fbioe.2020.00161 32211393 PMC7066983

[B31] Radoš D. Turner D. L. Catarino T. Hoffart E. Neves A. R. Eikmanns B. J. (2016). Stereospecificity of *Corynebacterium glutamicum* 2,3-Butanediol Dehydrogenase and Implications for the Stereochemical Purity of Bioproduced 2,3-Butanediol. Appl. Microbiol. Biotechnol. 100, 10573–10583. 10.1007/s00253-016-7860-6 27687994

[B32] Rochat D. Meillour P. N. L. Esteban-Duran J. R. Malosse C. Perthuis B. Morin J. P. (2000). Identification of Pheromone Synergists in American palm Weevil, *Rhynchophorus palmarum*, and Attraction of Related *Dynamis borassi* . J. Chem. Ecol. 26, 155–187. 10.1023/A:1005497613214

[B33] Ryu C. M. Farag M. A. Hu C. H. Reddy M. S. Wei H. X. Pare P. W. (2003). Bacterial Volatiles Promote Growth in *Arabidopsis* . Proc. Natl. Acad. Sci. U.S.A. 100, 4927–4932. 10.1073/pnas.0730845100 12684534 PMC153657

[B34] Salvachúa D. Werner A. Z. Pardo I. Michalska M. Black B. A. Donohoe B. S. (2020). Outer Membrane Vesicles Catabolize Lignin-Derived Aromatic Compounds in *Pseudomonas putida* KT2440. Proc. Natl. Acad. Sci. USA 117, 9302–9310. 10.1073/pnas.1921073117 32245809 PMC7196908

[B35] Sánchez-Pascuala A. Fernández-Cabezón L. de Lorenzo V. Nikel P. I. (2019). Functional Implementation of a Linear Glycolysis for Sugar Catabolism in *Pseudomonas putida* . Metab. Eng. 54, 200–211. 10.1016/j.ymben.2019.04.005 31009747

[B36] Schäfer A. Tauch A. Jäger W. Kalinowski J. Thierbach G. Pühler A. (1994). Small Mobilizable Multi-Purpose Cloning Vectors Derived from the *Escherichia coli* Plasmids pK18 and pK19: Selection of Defined Deletions in the Chromosome of *Corynebacterium glutamicum* . Gene 145, 69–73. 10.1016/0378-1119(94)90324-7 8045426

[B37] Sun J. Lu L. B. Liang T. X. Yang L. R. Wu J. P. (2020). CRISPR-assisted Multiplex Base Editing System in *Pseudomonas putida* KT2440. Front. Bioeng. Biotechnol. 8, 905. 10.3389/fbioe.2020.00905 32850749 PMC7413065

[B38] Taghavi S. van der Lelie D. Hoffman A. Zhang Y. B. Walla M. D. Vangronsveld J. (2010). Genome Sequence of the Plant Growth Promoting Endophytic Bacterium *Enterobacter* sp. 638. PLoS Genet. 6, e1000943. 10.1371/journal.pgen.1000943 20485560 PMC2869309

[B39] Takeda K. Matsumura H. Ishida T. Samejima M. Igarashi K. Nakamura N. (2013). The Two-step Electrochemical Oxidation of Alcohols Using a Novel Recombinant PQQ Alcohol Dehydrogenase as a Catalyst for a Bioanode. Bioelectrochemistry 94, 75–78. 10.1016/j.bioelechem.2013.08.001 24036413

[B40] Tiso T. Ihling N. Kubicki S. Biselli A. Schonhoff A. Bator I. (2020). Integration of Genetic and Process Engineering for Optimized Rhamnolipid Production Using *Pseudomonas putida* . Front. Bioeng. Biotechnol. 8, 976. 10.3389/fbioe.2020.00976 32974309 PMC7468518

[B41] Vu H. N. Subuyuj G. A. Vijayakumar S. Good N. M. Martinez-Gomez N. C. Skovran E. (2016). Lanthanide-dependent Regulation of Methanol Oxidation Systems in *Methylobacterium extorquens* AM1 and Their Contribution to Methanol Growth. J. Bacteriol. 198, 1250–1259. 10.1128/JB.00937-15 26833413 PMC4859578

[B42] Wada A. Prates É. T. Hirano R. Werner A. Z. Kamimura N. Jacobson D. A. (2021). Characterization of Aromatic Acid/proton Symporters in *Pseudomonas putida* KT2440 toward Efficient Microbial Conversion of Lignin-Related Aromatics. Metab. Eng. 64, 167–179. 10.1016/j.ymben.2021.01.013 33549838

[B43] Wehrmann M. Berthelot C. Billard P. Klebensberger J. (2018). The PedS2/PedR2 Two-Component System Is Crucial for the Rare Earth Element Switch in *Pseudomonas putida* KT2440. mSphere 3, e00376. 10.1128/mSphere.00376-18 30158283 PMC6115532

[B44] Wehrmann M. Billard P. Martin-Meriadec A. Zegeye A. Klebensberger J. (2017). Functional Role of Lanthanides in Enzymatic Activity and Transcriptional Regulation of Pyrroloquinoline Quinone-dependent Alcohol Dehydrogenases in *Pseudomonas putida* KT2440. mBio 8, e00570. 10.1128/mBio.00570-17 28655819 PMC5487730

[B45] Weimer A. Kohlstedt M. Volke D. C. Nikel P. I. Wittmann C. (2020). Industrial Biotechnology of *Pseudomonas putida*: Advances and Prospects. Appl. Microbiol. Biotechnol. 104, 7745–7766. 10.1007/s00253-020-10811-9 32789744 PMC7447670

[B46] Wu X. Monchy S. Taghavi S. Zhu W. Ramos J. van der Lelie D. (2011). Comparative Genomics and Functional Analysis of Niche-specific Adaptation in *Pseudomonas putida* . FEMS Microbiol. Rev. 35, 299–323. 10.1111/j.1574-6976.2010.00249.x 20796030 PMC3056050

[B47] Xiao Z. Lv C. Gao C. Qin J. Ma C. Liu Z. (2010). A Novel Whole-Cell Biocatalyst with NAD^+^ Regeneration for Production of Chiral Chemicals. PLoS One 5, e8860. 10.1371/journal.pone.0008860 20126645 PMC2811184

[B48] Xiao Z. Xu P. (2007). Acetoin Metabolism in Bacteria. Crit. Rev. Microbiol. 33, 127–140. 10.1080/10408410701364604 17558661

[B49] Xu Y. Chu H. Gao C. Tao F. Zhou Z. Li K. (2014). Systematic Metabolic Engineering of *Escherichia coli* for High-Yield Production of Fuel Bio-chemical 2,3-Butanediol. Metab. Eng. 23, 22–33. 10.1016/j.ymben.2014.02.004 24525331

[B50] Yang J. Im Y. Kim T. H. Lee M. J. Cho S. Na J. G. (2020). Engineering *Pseudomonas putida* KT2440 to Convert 2,3-Butanediol to Mevalonate. Enzyme Microb. Technol. 132, 109437. 10.1016/j.enzmictec.2019.109437 31731966

[B51] Yang T. Rao Z. Zhang X. Xu M. Xu Z. Yang S. T. (2017). Metabolic Engineering Strategies for Acetoin and 2,3-Butanediol Production: Advances and Prospects. Crit. Rev. Biotechnol. 37, 990–1005. 10.1080/07388551.2017.1299680 28423947

[B52] Yi H. S. Ahn Y. R. Song G. C. Ghim S. Y. Lee S. Lee G. (2016). Impact of a Bacterial Volatile 2,3-Butanediol on *Bacillus subtilis* Rhizosphere Robustness. Front. Microbiol. 7, 993. 10.3389/fmicb.2016.00993 27446033 PMC4923110

[B53] Zhang L. Xu Q. Zhan S. Li Y. Lin H. Sun S. (2014). A New NAD(H)-dependent *meso*-2,3-Butanediol Dehydrogenase from an Industrially Potential Strain *Serratia marcescens* H30. Appl. Microbiol. Biotechnol. 98, 1175–1184. 10.1007/s00253-013-4959-x 23666479

[B54] Zhang M. Gao C. Guo X. Guo S. Kang Z. Xiao D. (2018). Increased Glutarate Production by Blocking the Glutaryl-CoA Dehydrogenation Pathway and a Catabolic Pathway Involving L-2-Hydroxyglutarate. Nat. Commun. 9, 2114. 10.1038/s41467-018-04513-0 29844506 PMC5974017

[B55] Zhang M. Kang Z. Guo X. Guo S. Xiao D. Liu Y. (2019). Regulation of Glutarate Catabolism by GntR Family Regulator CsiR and LysR Family Regulator GcdR in *Pseudomonas putida* KT2440. mBio 10, e01570. 10.1128/mBio.01570-19 31363033 PMC6667623

[B56] Zhou Y. Lin L. Wang H. Zhang Z. Zhou J. Jiao N. (2020). Development of a CRISPR/Cas9n-based Tool for Metabolic Engineering of *Pseudomonas putida* for Ferulic Acid-to-Polyhydroxyalkanoate Bioconversion. Commun. Biol. 3, 98. 10.1038/s42003-020-0824-5 32139868 PMC7058019

