# Supplementary material for: Dehydrogenation Mechanism of Three Stereoisomers of Butane-2,3-Diol in Pseudomonas putida KT2440
Source: Front Bioeng Biotechnol. 2021 Aug 26;9:728767. doi: 10.3389/fbioe.2021.728767 (PMC8427195; doi:10.3389/fbioe.2021.728767)
Supplement: Supplementary file 1 [file DataSheet1.docx]

Supplementary Material

Dehydrogenation Mechanism of Three Stereoisomers of Butane-2,3-Diol in *Pseudomonas putida* KT2440

# Supplementary Figures and Tables

## 1. Supplementary Figures


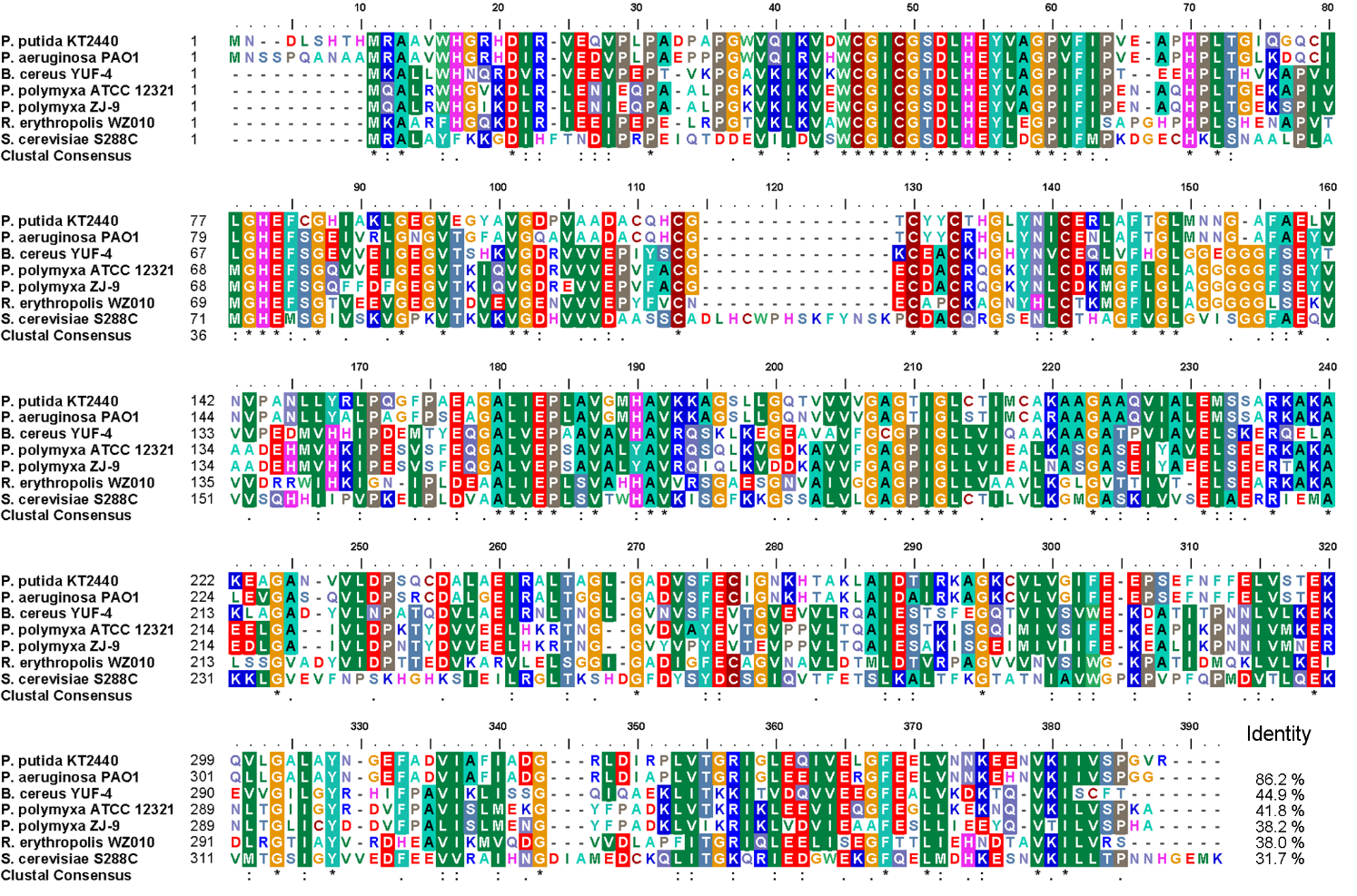


**Supplementary Figure S1**: Multiple sequence alignment of *R*,*R*-BDH in *P. putida* KT2440 with other reported *R*,*R*-BDHs in *P. aeruginosa* PAO1, *Bacillus cereus* YUF-4, *Paenibacillus polymyxa* ATCC 12321, *P. polymyxa* ZJ-9, *Rhodococcus erythropolis* WZ010, and *Saccharomyces cerevisiae* S288C. The alignment was performed using the BioEdit program. Detailed sequence information of different *R*,*R*-BDHs is listed in **Supplementary Table S3**.


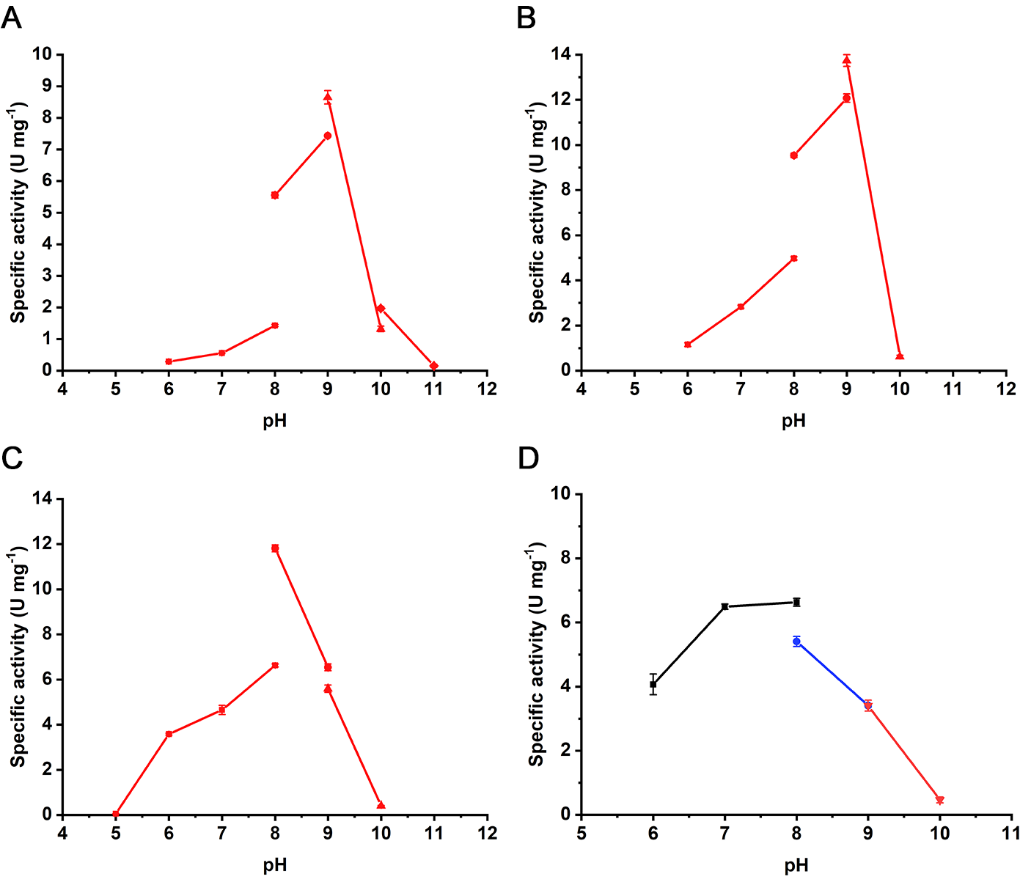


**Supplementary Figure S2**: The optimum pH and pH stability of *R*,*R*-BDH. The optimum pH for *meso*-2,3-BDO **(A)**, (2*R*,3*R*)-2,3-BDO **(B)**, and AC **(C)** were measured at 30℃ over the pH range from 4.0 to 10.0 with the following buffers: citrate-Na_2_HPO_4_ buffer (pH 4.0-8.0), Tris-HCl buffer (pH 8.0-9.0), and glycine-NaOH buffer (pH 9.0-10.0). **(D)** pH stability of *R*,*R*-BDH. The purified *R*,*R*-BDH was diluted with corresponding buffers and kept on ice for 30 min before determining its oxidative activity toward *meso*-2,3-BDO at 30℃. Error bars indicate deviation from the means (*n* = 3).


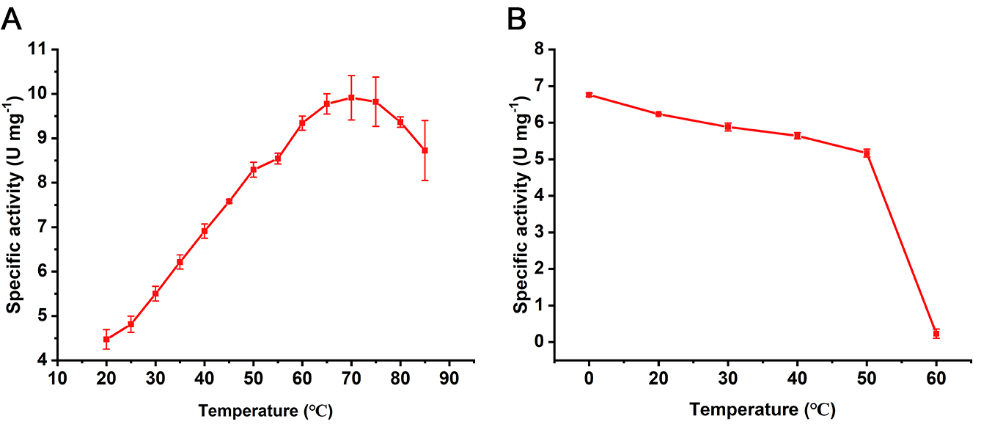


**Supplementary Figure S3**: The optimum temperature **(A)** and temperature stability **(B)** of *R*,*R*-BDH. The optimum temperature was determined by measuring the oxidative activity toward *meso*-2,3-BDO in Tris-HCl buffer (pH 7.4, 50 mM) equilibrated with thermostatic water bath at different temperatures previously. For temperature stability, the enzyme was incubated with metal bath at different temperature for 30 min and the enzymatic activity was determined by the oxidative activity toward *meso*-2,3-BDO. Error bars indicate deviation from the means (*n* = 3).


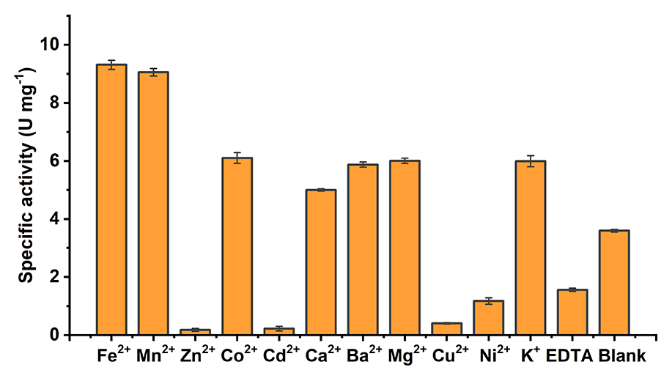


**Supplementary Figure S4**: The effects of metal ions on the enzymatic activity of *R*,*R*-BDH. The final concentration of different metal ions was 1 mM. The enzymatic activity was determined by measuring the oxidative activity of *R*,*R*-BDH toward *meso*-2,3-BDO in Tris-HCl buffer (pH 7.4, 50 mM) at 30℃. A blank was conducted without adding any metal ions. Error bars indicate deviation from the means (*n* = 3).


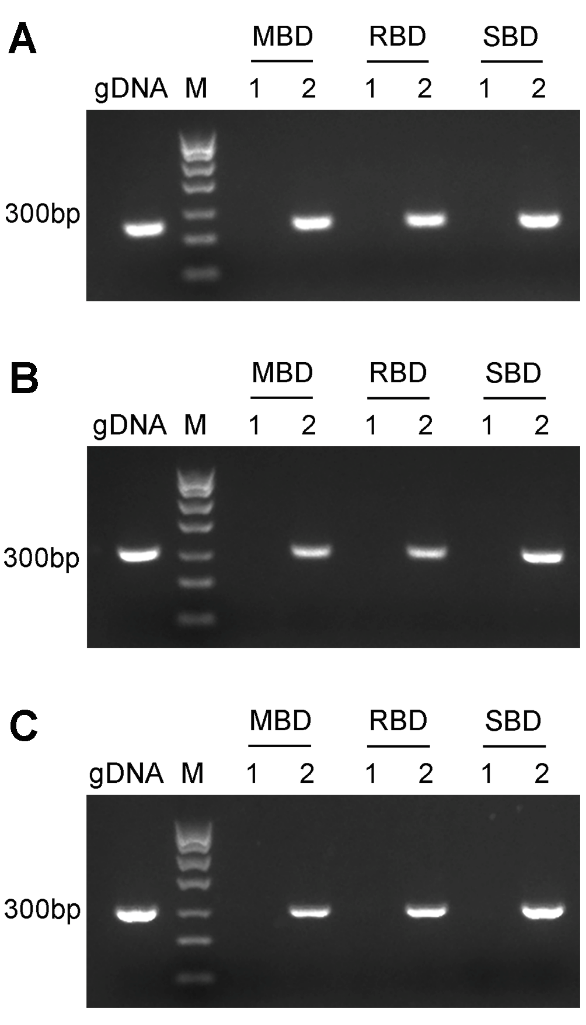


**Supplementary Figure S5**: Analysis of the transcription of *pp0552* **(A)**, *pedE* **(B)** and *pedH* **(C)** during 2,3-BDO utilization of *P. putida* KT2440 by RT-PCR (lane 2). The genomic DNA (gDNA) and RNA (lane 1) of *P. putida* KT2440 were used as positive and negative control, respectively. *P. putida* KT2440 were cultured in MSM with 2 g L^-1^ *meso*-2,3-BDO (MBD), (2*R*,3*R*)-2,3-BDO (RBD), or (2*S*,3*S*)-2,3-BDO (SBD) as the sole carbon source.

## 2. Supplementary Tables

Supplementary Table S1: Strains and vectors used in this work.

| Strain or plasmid | Characteristic(s) | Source or reference |
| --- | --- | --- |
| Strains | | |
| *Pseudomonas putida* | | |
| KT2440 | Wild-type | ATCC |
| Δ*pp0552* | *P. putida* KT2440 *pp0552* disruption mutant strain | This work |
| Δ*pedH* | *P. putida* KT2440 *pedH* (*pp2679*) disruption mutant strain | This work |
| Δ*pedE* | *P. putida* KT2440 *pedE* (*pp2674*) disruption mutant strain | This work |
| Δ*pp0552*Δ*pedH* | *P. putida* KT2440 *pp0552* and *pedH* disruption mutant strain | This work |
| Δ*pp0552*Δ*pedE* | *P. putida* KT2440 *pp0552* and *pedE* disruption mutant strain | This work |
| Δ*pp0552*Δ*pedH*Δ*pedE* | *P. putida* KT2440 *pp0552*, *pedH*, and *pedE* disruption mutant strain | This work |
| *E. coli* | | |
| DH5α | F^-^ φ80d*lac*ZΔM15 Δ(*lacZYA*-*argF*)*U169* *recA1* *endA1* *hsdR17*(r_K_^-^ m_K_^-^) *supE44* *thi-1* *gyrA* *relA1*; used for plasmids clone | Invitrogen |
| BL21 | F^-^ *ompT* *gal* *dcm* *lon* *hsdS*_B_(r_B_^-^ m_B_^-^) λ(DE3) *pLysS*(cm^r^); used for protein expression | Invitrogen |
| BL21-PP0552 | BL21 harboring the expression plasmid pETDuet-*pp0552* (*P. putida* KT2440) | This work |
| Plasmid | | |
| pK18*mobsacB* | *ori*ColE1 Mob^+^, *lacZα*; used for directed insertional disruption | Schäfer et al., 1994 |
| pK18*mobsacB*-Δ*pp0552* | Partial lengthes of *pp0552* were inserted into pK18*mobsacB* | This work |
| pK18*mobsacB*-Δ*pedH* | Partial lengthes of *pedH* were inserted into pK18*mobsacB* | This work |
| pK18*mobsacB*-Δ*pedE* | Partial lengthes of *pedE* were inserted into pK18*mobsacB* | This work |
| pETDuet-1 | Ap^r^, vector for protein expression | Novagen |
| pETDuet-*pp0552* | Ap^r^, pETDuet-1 with *pp0552* gene of *P. putida* KT2440 | This work |

Supplementary Table S2: Primers used in this work.

| Primer name | Sequence |
| --- | --- |
| *For gene knockout* |  |
| *pp0552*-up-F | 5'-GGATCCATGAATGACCTGAG-3' |
| *pp0552*-up-R | 5'-AGGACATCTCAAGGCAGCTCGGCGAAGG-3' |
| *pp0552*-down-F | 5'-CCTTCGCCGAGCTGCCTTGAGATGTCCT-3' |
| *pp0552*-down-R | 5'-CTGCAGTCAGCGCACACCTGGT-3' |
| *pedH*-up-F | 5'-GGATCCATGACCCGATCCCCACGT-3' |
| *pedH*-up-R | 5'-TGTCCAGGCCAGTGATAGCCCATGTGC-3' |
| *pedH*-down-F | 5'-GCACATGGGCTATCACTGGCCTGGACA-3' |
| *pedH*-down-R | 5'-AAGCTTTTATGGCTTGACGCTTGC-3' |
| *pedE*-up-F | 5'-GGATCCATGACAATAAGATCGCTACC-3' |
| *pedE*-up-R | 5'-TCAACCACATAGAAGCATCCACACTTCTTC-3' |
| *pedE*-down-F | 5'-GAAGAAGTGTGGATGCTTCTATGTGGTTGA-3' |
| *pedE*-down-R | 5'-AAGCTTTCAACGTTGTGCAGTC-3' |
| *For protein expression* | |
| *pp0552*-F | 5’-GGATCCAATGAATGACCTGAGC-3’ |
| *pp0552*-R | 5’-CTTAAGTCAGCGCACACCTGGT-3’ |
| *For cotranscription assay* | |
| (*acoR-acoX*)-F | 5’-GCCAGAGGCAGGGTTGGCAATGATC-3’ |
| (*acoR-acoX*)-R | 5’-TTCAACACCCTGCTGACGCAGTCCA-3’ |
| (*acoX-acoA*)-F | 5’-AATCGAATTTGCCGAGCATGACACG-3’ |
| (*acoX-acoA*)-R | 5’-ATACAGGTGGACGAAGCCGGGAATT-3’ |
| (*acoA-acoB*)-F | 5’-CCGCGTCGAGGACCTGATCGAAGAC-3’ |
| (*acoA-acoB*)-R | 5’-CCGGCGACGTCTTCGCCAATGATGA-3’ |
| (*acoB-acoC*)-F | 5’-GCGCACTCAAGGGCCCGATCGAAAT-3’ |
| (*acoB-acoC*)-R | 5’-TGAAAGGCGCTTCGACGCTGCTGCT-3’ |
| (*acoC-pp0552*)-F | 5’-GGAAGCCGAAGTACAGGTGCTGCCA-3’ |
| (*acoC-pp0552*)-R | 5’-CACCAGTCCACCTTGATCTGCACCC-3’ |
| *For transcription analysis* |  |
| *pp0552*-RT-PCR-F | 5’-ATCGGCAACAAACATACGG-3’ |
| *pp0552*-RT-PCR-R | 5’-GCTCGACAATCTGCTCCA-3’ |
| *pedE*-RT-PCR-F | 5’-CCGCAACGGTTTCTTCTAT-3’ |
| *pedE*-RT-PCR-R | 5’-GTTCACTTCCTCGGTCCAG-3’ |
| *pedH*-RT-PCR-F | 5’-CTGCGACGTAATCAACCG-3’ |
| *pedH*-RT-PCR-R | 5’-TAGCCCATGTGCCCTTCC-3’ |

Supplementary Table S3: Kinetic parameters of different *R*,*R*-BDHs toward *meso*-2,3-BDO and (2*R*,3*R*)-2,3-BDO dehydrogenation.

| Source | Protein accession | (2*R*,3*R*)-2,3-BDO | | *meso*-2,3-BDO | | References |
| --- | --- | --- | --- | --- | --- | --- |
|  |  | *K*_m_ (mM) | *K*_cat_/*K*_m_  (s^-1^···mM^-1^) | *K*_m_ (mM) | *K*_cat_/*K*_m_ (s^-1^···mM^-1^) |  |
| *Pseudomonas putida* KT2440 | NP_742715.1 | 0.155 ± 0.016 | 154.186 ± 9.227 | 0.251 ± 0.022 | 84.328 ± 5.799 | This work |
| *Pseudomonas aeruginosa* PAO1 | NP_252842.1 | 0.057 ± 0.004 | 12.71 ± 0.072 | 0.128 ± 0.010 | 6.098 ± 0.068 | Liu et al., 2018 |
| *Rhodococcus erythropolis* WZ010 | AKE50911.1 | 0.58 ± 0.05 | 7.379 ± 0.86 | NR | NR | Yu et al., 2015 |
| *Paenibacillus polymyxa* ATCC 12321 | ADV15558.1 | 1.76 ± 0.29 | NR | 5.62 ± 0.81 | NR | Yu et al., 2011 |
| *Bacillus cereus* YUF-4 | BAB60856.1 | 6.9 | NR | 10.35 | NR | Hosaka et al., 2001 |
| *Saccharomyces cerevisiae* S288C | NP_009341.2 | 14 ± 5 | 93 ± 33 | 65 ± 9 | 12 ± 2 | González et al., 2000 |
| *Paenibacillus polymyxa* ZJ-9 | AEV53933.1 | 7.67 ± 0.73 | NR | 2.73 ± 0.22 | NR | Gao et al., 2012 |

NR, not reported

**References**

Gao, J., Yang, H. H., Feng, X. H., Li, S., Xu, H. (2013). A 2,3-butanediol dehydrogenase from *Paenibacillus polymyxa* ZJ-9 for mainly producing *R*,*R*-2,3-butanediol: purification, characterization and cloning. *J. Basic Microbiol.*, 53, 733–741. doi: 10.1002/jobm.201200152

González E., Fernández M.R., Larroy C., Solà L., Pericàs M.A., Parés X., et al. (2000). Characterization of a (2*R*,3*R*)-2,3-butanediol dehydrogenase as the *Saccharomyces cerevisiae* *YAL060W* gene product. Disruption and induction of the gene. *J. Biol. Chem.*, 275, 35876–35885. doi: 10.1074/jbc.M003035200.

Hosaka T., Ui S., Ohtsuki T., Mimura A., Ohkuma M., Kudo T. (2001). Characterization of the NADH-linked acetylacetoin reductase/2,3-butanediol dehydrogenase gene from *Bacillus cereus* YUF-4. *J. Biosci. Bioeng.*, 91, 539–544. doi:10.1263/jbb.91.539

Liu, Q., Liu, Y., Kang, Z., Xiao, D., Gao, C., Xu, P., et al. (2018). 2,3-Butanediol catabolism in *Pseudomonas aeruginosa* PAO1. *Environ. Microbiol.*, 20, 3927−3940. doi: 10.1111/1462-2920.14332

Yu B., Sun J., Bommareddy R.R., Song L., Zeng A.P. (2011). Novel (2*R*,3*R*)-2,3-butanediol dehydrogenase from potential industrial strain *Paenibacillus polymyxa* ATCC 12321. *Appl. Environ. Microbiol.*, 77, 4230–4233. doi:10.1128/AEM.02998-10

Yu M., Huang M., Song Q., Shao J., Ying X. (2015). Characterization of a (2*R*,3*R*)-2,3-butanediol dehydrogenase from *Rhodococcus erythropolis* WZ010. *Molecules*, 20, 7156–7173. doi:10.3390/molecules20047156

Schäfer, A., Tauch, A., Jäger, W., Kalinowski, J., Thierbach, G., and Pühler, A. (1994). Small mobilizable multi-purpose cloning vectors derived from the *Escherichia coli* plasmids pK18 and pK19: selection of defined deletions in the chromosome of *Corynebacterium glutamicum*. Gene, 145, 69–73. doi: 10.1016/0378-1119(94)90324-7
